# Variations of intact phospholipid compositions in the digestive system of Antarctic krill, *Euphausia superba*, between summer and autumn

**DOI:** 10.1371/journal.pone.0295677

**Published:** 2023-12-29

**Authors:** Simone Heyen, Vivien Schneider, Lukas Hüppe, Bettina Meyer, Heinz Wilkes

**Affiliations:** 1 Institute for Chemistry and Biology of the Marine Environment (ICBM), Carl von Ossietzky University of Oldenburg, Oldenburg, Germany; 2 Alfred Wegener Institute for Polar and Marine Research, Bremerhaven, Germany; 3 Julius-Maximilians-University of Würzburg, Würzburg, Germany; 4 Helmholtz Institute for Marine Functional Biodiversity (HIFMB), Carl von Ossietzky University of Oldenburg, Oldenburg, Germany; University of Shiga Prefecture, JAPAN

## Abstract

The biochemical composition of Antarctic krill, *Euphausia superba*, is largely determined by their feeding behaviour. As they supply energy for animals of a higher trophic level and are also commercialized for human consumption, the interest in research on the species is high. Lipids, especially phospholipids, make up a high proportion of dry weight in krill. Seasonal changes are well documented in the fingerprint of free fatty acids analysed after hydrolysis of phospholipids, but the underlying intact polar lipids are rarely considered. In this study, we evaluated the compositions of intact phospholipids (IPLs) in the stomach, digestive gland and hind gut of Antarctic krill caught in summer and autumn at the Antarctic Peninsula region. Using high-resolution mass spectrometry, the fatty acid composition of 179 intact phospholipids could be resolved. Most IPLs were phosphatidylcholines, followed by phosphatidylethanolamines. Several very long chain polyunsaturated fatty acids up to 38:8, which have not been reported in krill before, were identified. The composition shifted to higher molecular weight IPLs with a higher degree of unsaturation for summer samples, especially for samples of the digestive gland. The data supplied in this paper provides new insights into lipid dynamics between summer and autumn usually described by free fatty acid biomarkers.

## Introduction

The ecosystem of the Southern Ocean depends on the availability of Antarctic krill (*Euphausia superba*, hereafter krill) as central organism of the food web, being a primary food source for many marine animals, such as whales, penguins, or seals and contributing to the nutrient cycle in these waters [1]. Because of the krills ubiquitous circumpolar abundance, their enormous biomass of about 379 million tonnes and their importance in biogeochemical cycles, krill is one of the most studied pelagic macrozooplankton species [2]. However, krill densities in the circumpolar region are declining and the ongoing climate change will further alter their habitat, food availability and reproductivity [3–5]. Therefore, more research is needed to enhance the knowledge on drivers for physiological changes affecting the krill population.

Lipids can account for over 40% of dry mass in krill, forming a considerably large fatty acid repository, which can be subject to alterations due to external influences, such as the dietary input driven by seasonal variations of phytoplankton abundance [6, 7]. The major lipid classes found in krill are triacylglycerols accounting for around 50% and phospholipids accounting for 40% or more of the total lipid content [7–9]. The latter are not only the primary cell membrane constituents but also serve as energy storage in krill, especially the subclass of phosphatidylcholines [10]. While the fatty acid composition of lipids after hydrolysis is frequently used to evaluate spatial or temporal variability, one of the lesser studied fields of krill research are intact phospholipids (IPLs) [11–13]. So far, the composition of IPLs has only been studied in krill oil, examining its nutritional value [14–16]. Krill oil attracts substantial interest due to its numerous health-related beneficial effects, therefore being useful for medical applications [17–19]. The phospholipid fraction from krill oil is administered as a dietary supplement due to an enhanced bioavailability compared to triacylglycerols in fish oil, even though other factors like the food matrix or the amount of free fatty acids in the krill oil may also be responsible for a better absorption [20–22].

With the advances in high-resolution mass spectrometry, very low-abundant lipids can be detected with high sensitivity and selectivity [23, 24]. In contrast to the often applied gas chromatographic analyses for the determination of the overall fatty acid composition of certain lipid classes, liquid chromatographic-tandem mass spectrometric experiments enable the evaluation of the sum formulas of intact lipids as well as of the fatty acid compositions of these intact lipid species [25]. This is especially useful for detecting lipids containing very long chain fatty acids, which are rarely considered in studies conducted on the fatty acid composition of krill, as those are very low in abundance and hard to detect during gas chromatographic measurements [9].

Fatty acids are used as biomarkers to investigate dietary differences in krill [11, 26, 27]. Earlier studies have shown that the digestive gland functions as a significant repository for fatty acids, revealing trophic signals [28, 29]. Of the three essential omega-3-fatty acids eicosapentaenoic acid (EPA, 20:5), docosahexaenoic acid (DHA, 22:6) and α-linolenic acid (ALA, 18:3), EPA and DHA are very abundant fatty acids in krill and predominantly taken up from food [6, 8]. However, accumulation of these polyunsaturated fatty acids (PUFAs) in the digestive gland was demonstrated by Virtue et al., who found levels of EPA and DHA that were higher than what could have possibly been obtained by the phytoplankton diet provided, concluding that they originated from the alteration of other fatty acids occurring in this organ [28].

PUFAs possess several health benefits, fuelling the commercial interest in krill [30]. Notably, krill fishery has steadily increased since 1993, especially in the Southwest Atlantic sector of the Southern Ocean [31]. The Commission for the Conservation of Antarctic Marine Living Resources (CCAMLR) monitors and manages fishing rates and sets annual catchment limits in order to maintain the current status of the krill population. To be able to detect and evaluate future changes and impacts on the krill population, it is crucial to enhance the repertoire of available tracers documenting the biogeochemical status.

In this study, we have investigated the variability in the composition of IPLs between summer and autumn and along the digestive system. Special focus was set on the detection of phospholipids containing very long chain polyunsaturated fatty acids (VLCPUFAs) not considered in krill before. This was achieved by applying highly sensitive high-resolution mass spectrometry during which the identification of intact phospholipids was based on accurate masses and characteristic fragment ions occurring during positive and negative electrospray ionization.

## Materials and methods

### Krill samples

Krill were collected during two cruises (03.05.-17.06.2021 and 02.01.-26.03.2022) on board the commercial krill fishing vessel (FV) *Antarctic Endurance* of the company Aker BioMarine. The *Antarctic Endurance* is a beam trawler, equipped with a continuous fishing system, which uses a vacuum system to continuously pump freshly caught krill from the cod ends of the two nets on board (trawl speed ~ 2 knots). When entering the ship, the krill is separated from excess water on a metal grid, from which krill can be sampled. Freshly caught krill were taken from the continuous fishing system on 13^th^ and 31^st^ May (autumn) of 2021 in the Bransfield Strait (62.497°S; 57.290°W and 62.581°S; 57.440°W, respectively) and on 19^th^ January and 6^th^ March (summer) of 2022 at the South Orkney Islands (60.101°S, 46.179°W and 60.147°S, 46.145°W, respectively). Originating from rather moderate regions of Antarctica in terms of seasonal extremes, these areas are influenced by transitional periods between Antarctic summer and winter. As austral spring and autumn are important feeding periods for *Euphausia superba*, these transitional phases were also considered during our analysis.

**Fig 1:**
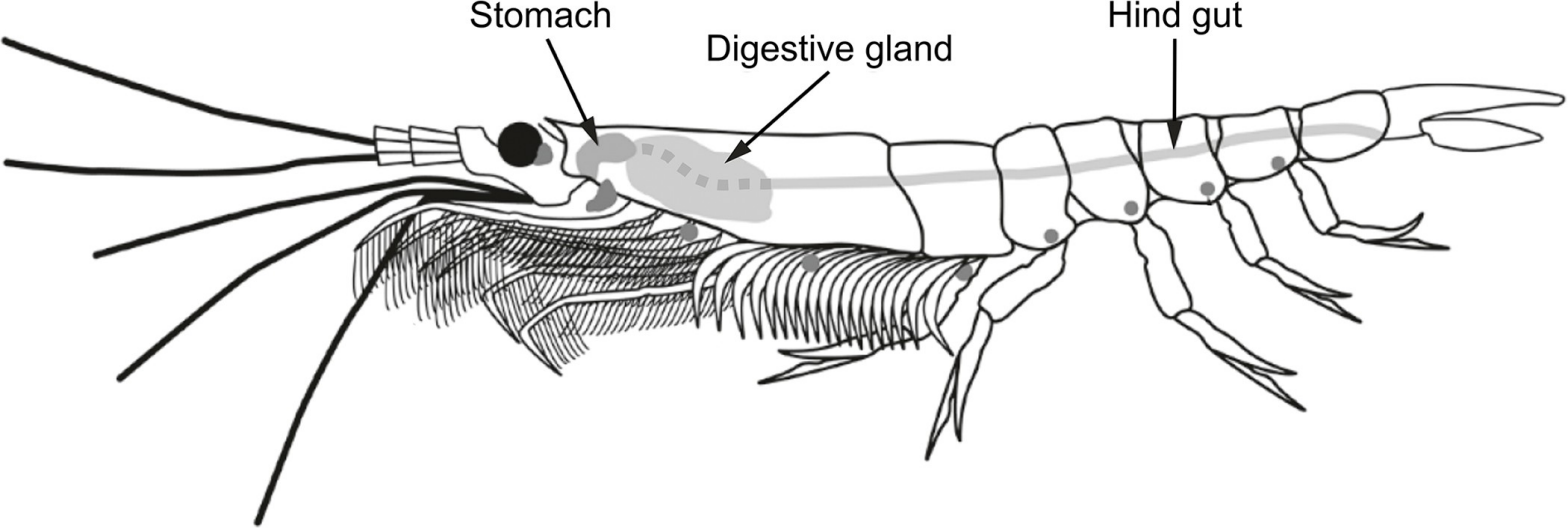
Anatomical locations of the organs sampled from Antarctic krill, *Euphausia superba*. Modified after The Curious Life of Krill by Stephen Nicol (Drawing by Marcia Rackstraw). Copyright © 2018 Stephen Nicol. Reproduced by permission of Island Press, Washington, DC [32].

The stomach, digestive gland and hindgut of krill individuals, as depicted in Fig 1, were dissected on board under a stereomicroscope (Leica M125 C, Leica Microsystems GmbH, Wetzlar, Germany) directly after sampling. The three organs of the digestive system were separated and immediately frozen and stored at ₋80°C until further analysis. Samples from individual krill specimens were used to analyse IPLs and free fatty acids. Eight to twelve replicates for every sampling time and organ were used for the analysis. Additionally, samples from three krill individuals from each sampling time and from which the digestive system was removed were used to determine the lipid signatures independent of the digestive system.

### Extraction

Sample extraction was performed under dark conditions using minimal artificial light to prevent possible isomerization of the lipids [33]. To avoid autolytic processes, samples were transported on dry ice between extraction steps [34]. Initial extraction of lipids from the krill matrix was done applying a bead-beating protocol with methanol. For this, the tissues were transferred into cryotubes filled with 0.5 g of 0.1 mm glass beads, 0.7 g of 0.7 mm zirconia beads and 1 mL of methanol (MeOH). 40 *μ*L of a 1 mg/mL 1,2-distearoyl-*d*_70_-*sn*-glycero-3-phosphocholine solution was added as internal standard prior to extraction. Bead-beating was performed in three cycles of 30 seconds with an intermission on dry ice of 90 seconds to prevent thermal decomposition of the lipids. After centrifugation for 10 min at 12000 rpm, the supernatant was transferred into a separation funnel already containing 3 mL dichloromethane (DCM) and 2.1 mL ultrapure water. 0.5 mL of MeOH were added into the cryotube two more times, and the sample was vortexed and centrifuged again before also transferring the supernatant into the separation funnel. The complete bead-beating and centrifugation process was carried out three times, resulting in a total extraction volume of 6 mL, which were added to the separation funnel.

Subsequently, lipids were extracted based on a modified Bligh and Dyer extraction protocol previously applied for krill samples [35, 36]. After shaking the separation funnel, 3 mL DCM and 3 mL saline ultrapure water were added, changing the ratio of MeOH:DCM:H_2_O from 2:1:0.7 to 1:1:0.85. The lower layer acquired after phase separation was drained into an evaporation glass. In order to enhance the separation efficiency, this extraction step was repeated two times by adding DCM again. For an additional removal of interfering matrix compounds, the sample was refilled into the separation funnel after discarding the remaining water phase, 3 mL ultrapure water were added, the sample was shaken and the organic layer was drained into the evaporation glass after phase separation. Finally, the organic phase was evaporated under a gentle stream of nitrogen and stored at ₋20°C until further analysis. In parallel, blanks were processed similarly. Directly before the instrumental analyses, samples were resuspended in 1 mL DCM.

### Instrumental analyses

#### Liquid chromatography-high-resolution mass spectrometry of intact phospholipids

Before injection, 20 *μ*L of each sample was diluted in 180 *μ*L DCM:MeOH 4:5 (v/v). Chromatographic separation was achieved using a Vanquish Flex UHPLC (Thermo Scientific, Bremen, Germany), equipped with a Nucleoshell HILIC column (Macherey-Nagel, Düren, Germany). The column had a length of 150 mm, an inner diameter of 2 mm and a particle size of 2.7 *μ*m. Eluent A consisted of a 10 mM ammonium acetate buffer at pH 6, eluent B had the same buffer strength in acetonitrile and water (95:5 v:v). At a flow rate of 0.3 mL/min, the gradient started at 100% B held for 2.25 min, decreased to 50% B until 19.15 min, where it was held again for 1.5 min. After that, B increased back to 100% in 7.5 min, where it stayed for 15.55 min. The total runtime was 43.7 min. Retention times of IPLs were between 3.5 and 9 min, depending on the class. High-resolution mass spectrometric data was acquired on an Orbitrap Fusion mass spectrometer (Thermo Scientific, Bremen, Germany). Instrument settings for the different experiments are summarized in the S1 Table. For the identification of the phospholipid class, MS^2^ experiments in positive electrospray ionization mode (ESI+) were applied, triggering either the neutral loss of the head group (PE, PME, PDME, PS, PG and PI (full names listed in Table 1)) or the splitting off of the head group as characteristic ion (PC) as listed in Table 1. Fragmentation was induced by higher energy C trap dissociation. With negative electrospray ionization (ESI-) MS^2^ experiments, additional characteristic fragment ions as well as ions originating from the fatty acid side chains were observed after confirmation of the correct corresponding accurate mass of the intact molecular ion in MS^1^. For the quantification of IPLs, an ESI+ full scan was additionally measured to enhance the resolution of the peak. Due to identical retention times, isomers were integrated as combined peaks. The fatty acid compositions of isomers were identified based on the number and possible combinations of ESI- MS^2^ signals.

**Table 1:** Characteristic fragment ion and neutral loss masses indicative for all phospholipid classes analysed in this study for both positive and negative electrospray ionization.

Phospholipid	Characteristic signals in	
	ESI+ (*m/z*)	ESI- (*m/z*)
Phosphatidylcholine (PC)	184.0733	60.0211 NL
Phosphatidylethanolamine (PE)	141.0191 NL	
Phosphatidyl-*N*-methylethanolamine (PME)	155.0347 NL	
Phosphatidyl-*N*,*N´*-dimethylethanolamine (PDME)	169.0504 NL	
Phosphoinositide (PI)	277.0563 NL	259.0224
Phosphatidylglycerol (PG)	189.0402 NL	
Phosphatidylserine (PS)	185.0089 NL	87.0320 NL

NL, neutral loss difference

#### Gas chromatography-mass spectrometry of free fatty acids

Prior to measurements, 25 *μ*L from the total lipid extract were diluted in 25 *μ*L DCM and then hydrolysed and methylated for two hours at 70°C with 50 *μ*L trimethylsulfonium hydroxide. Gas chromatographic-mass spectrometric analysis was executed based on the method described in Heyen et al. on a Trace GC Ultra gas chromatograph coupled to an ISQ QD mass spectrometer (all from Thermo Scientific, Bremen, Germany) [37]. The gas chromatograph was equipped with a TriPlus autosampler, a PTV injector operated in splitless mode and an Agilent J&W DB-5 capillary column with a length of 30 m, an inner diameter of 0.25 mm and a film thickness of 0.25 *μ*m. The starting temperature of the oven was 60°C, which was held for two min and then raised to 325°C at 10 K/min, where the temperature was held for additional 15 min. The transfer line was set to 290°C and the ion source to 240°C. Electron impact ionization was performed at 70 eV. Full-scan mass spectra were obtained between *m/z* 50 and 650 at a scan time of 0.2 s. Standards for the identification were purchased from Sigma Aldrich (Steinheim, Germany), except for dodecanoic and heptadecanoic acid, which were from Merck (Darmstadt, Germany). Fatty acids for which no standard was available were identified based on fragmentation patterns in comparison with the NIST (National Institute of Standards and Technology) database and retention orders as listed in S2 Table.

#### Data analysis

Integration of peaks was performed using the Xcalibur Quan Browser, version 4.1.31.4 (Thermo Scientific, Bremen, Germany). For quantitative IPL data, calibrations between 0.1 pg/mL and 10 *μ*g/mL were prepared in quadruplicates using the standards PC(16:0), PE(16:0), PG(16:0) and PS(16:0), all with the addition of 4 *μ*g/mL of internal standard. After elimination of outliers applying the Grubbs test with a significance threshold of 0.05 and subtraction of process blanks from the samples, the standard PC(16:0) was used for the calibration of PC, PE(16:0) for PE, PME and PDME, PG(16:0) for PG and PI and PS(16:0) for PS. For each area ratio of an IPL to the internal standard in a sample, five calibration levels were chosen to calculate the concentration.

In case of the fatty acid calibration for the GC-MS measurements, fatty acids 22:0, 22:1 and 20:5 were used for the calibration of saturated, monounsaturated and polyunsaturated fatty acids, respectively. The deuterated 18:0 originating from the hydrolysis of the internal standard during derivatization was applied to correct for extraction and measurement uncertainties. Calibrations were prepared between 0.075 and 25 *μ*g/mL similar to the IPL calibration. Principal component analysis of relative IPL abundances was performed in R version 4.2.2, with the package ggbiplot used for visualisation [38, 39]. For the visualization of bar plots, the package ggplot2 was used [40].

As weighing of the samples without causing significant alterations due to autolytic processes was not feasible, only percentage distributions of IPLs and fatty acids in a sample were used for the following evaluations. Having the three variables month, sex and organ, we evaluated all seven possible combinations. During this process, we did not observe any trends based on sex. Therefore, this variable is not further considered below.

## Results and discussion

### Identification of intact phospholipids

**Fig 2:**
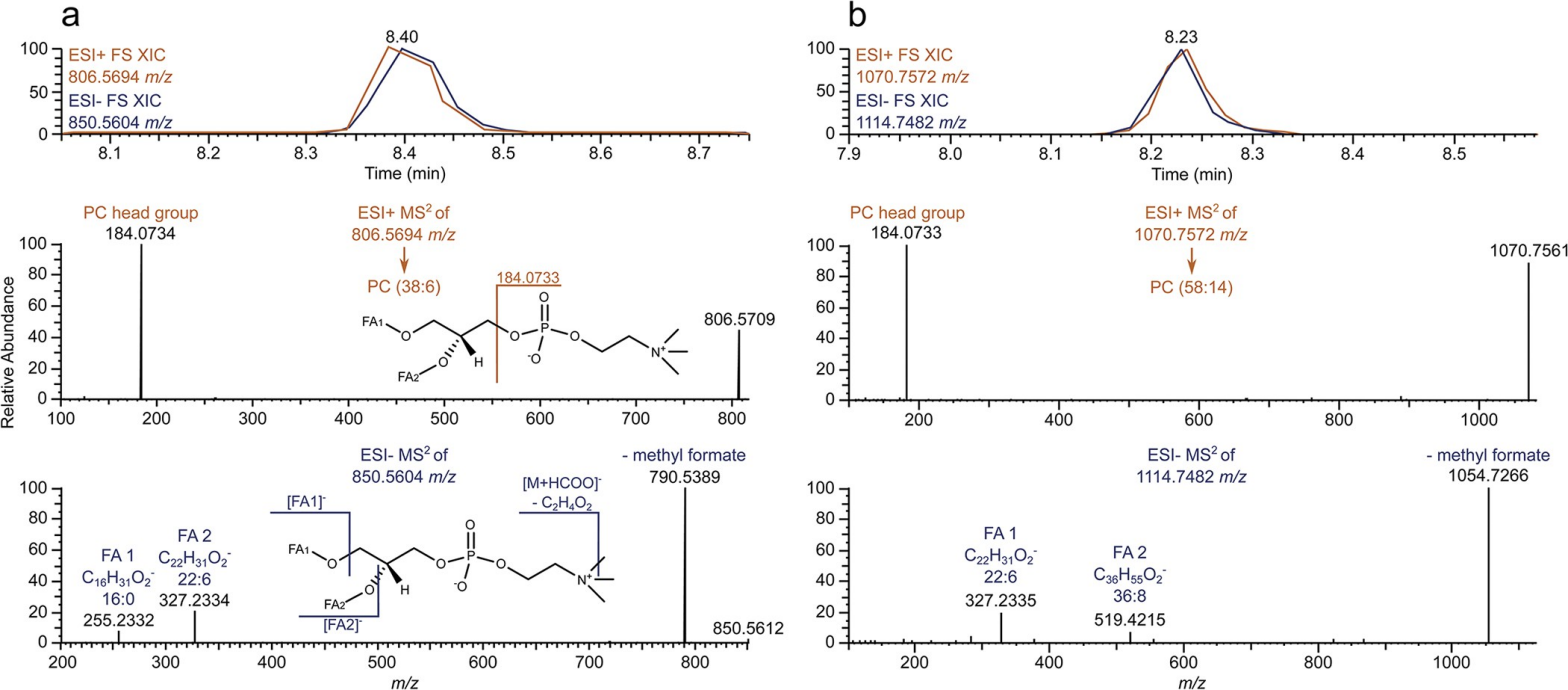
High-resolution mass spectrometry-based structural identification of intact phospholipids. a. Measured standard solution of an IPL (PC(38:6)) with 16:0 and 22:6 as the fatty acids in *sn*-1 and *sn*-2 position. b. Unknown IPL detected in samples identified as PC(58:14). Characteristic traits measured in ESI+ are coloured orange, whereas they are coloured blue for ESI-. In positive mode, the head group of the PC can be detected as ion. The exact mass of the [M+H]^+^ ion is used to denote the overall composition of the IPL. For PCs, an [M+HCOO]^-^ ion in ESI- can be expected to occur at the same retention time as the [M+H]^+^ ion in ESI+. However, for PCs, the most abundant ion in MS^2^ will be the one formed due to the neutral loss of methyl formate. Additionally, in ESI-, fatty acids can be assigned by finding their ion signals in the lower mass range. In the case of the unknown IPL depicted in b, 22:6 and 36:8 fatty acid signals were identified. The combination of these two fatty acids has to match the overall composition determined in ESI+.

For the identification of IPLs, only peaks occurring in both ESI+ and ESI- and being confirmed by additional characteristic MS^2^ fragment ions, as well as matching fatty acid signals, were considered. In total, 127 unique elemental compositions representing sum formulas of IPLs were identified. In some cases, the fragmentation patterns observed in ESI- MS^2^ implied the occurrence of more than one possible fatty acid combination for a single IPL sum formula, resulting in an overall number of 179 individual IPLs, not considering stereoisomers not distinguishable with the method applied. Most IPLs were PCs with 59 sum formulas (84 fatty acid combinations), followed by PEs with 27 (43), PDMEs with 13 (15), PIs with 11 (18), PMEs with 7 (8), PGs with 6 (6) and PSs with 4 (5) sum formulas, respectively. Indeed, PCs are the most abundant phospholipids in krill [41]. Earlier studies on krill oil identified a maximum of 69 PLs, almost exclusively being PCs and also including lyso- and ether PLs, which were not considered in our study [15, 19]. With the fatty acid signals found in ESI- MS^2^ spectra, we were able to identify several VLCPUFAs never reported in krill before, including a fatty acid with 36 carbon atoms and 8 double bond equivalents (DBE) along the chain. A fatty acid of this type has been identified as the unusual omega-3-fatty acid (all-*Z*)-hexatriaconta-12,15,18,21,24,27,30,33-octaenoic acid in the dinoflagellate *Amphidinium carterae* [42]. As we were not able to detect any of these high molecular weight fatty acids during GC-MS measurements, we did not have the option to use derivatisation techniques to confirm and locate the double bonds on the fatty acid chain. Therefore, ring structures can possibly be present in the side chains. Nevertheless, we will be labelling the fatty acids as number of carbons:DBEs in side chain (e.g. 36:8 for the above mentioned fatty acid). Fig 2 illustrates the high-resolution mass spectrometry-based structural identification of one of the PCs containing a VLCPUFA.

During LC-MS measurements of IPLs, 45 different fatty acids were identified, ranging from 12:0 to 36:8. The predominant fatty acids by number of occurrences were 20:5, 18:1, 22:6 and 16:0. The high abundance of especially 20:5 (EPA) and 22:6 (DHA) is in accordance with free fatty acid profiles in krill, as those fatty acids predominantly occur in phospholipids [6, 8, 12]. Another fatty acid appearing in multiple IPLs was 28:8. This fatty acid can be a marker for dinoflagellates and has, like many of the VLCPUFAs, not often been considered in studies of the fatty acid profiles of krill [9, 43, 44].

Regarding the abundances of the two fatty acid signals, it can be assumed that the more intense fragment ion represents the fatty acid at the *sn*-2 position of the IPL, even though this does depend on the MS settings [45, 46]. NMR experiments have determined that PUFAs in phospholipids of krill are present both at the *sn*-1 and *sn*-2 position, although *sn*-2 is predominantly represented [47]. When assuming that the more abundant fatty acyl fragment ion occurring in ESI- MS^2^ experiments represents the fatty acid at the *sn*-2 position, this is in accordance with our data [45, 46]. The complete list with all identified phospholipids, their fatty acid combinations and exact masses in ESI+ and ESI- can be found in S3 to S5 Tables.

### Variations in the intact phospholipid compositions between summer and autumn

**Fig 3:**
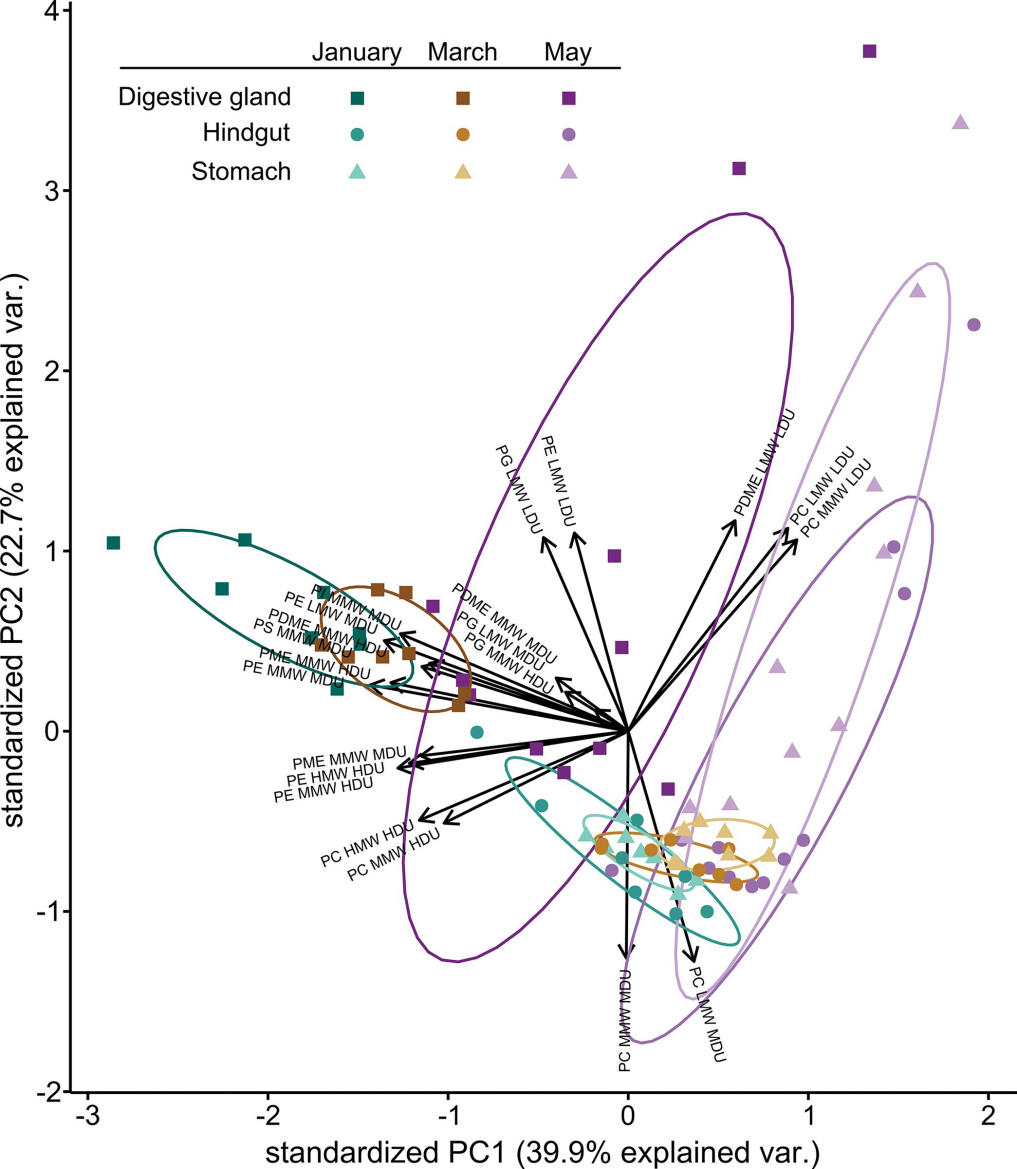
Principal component analysis of the percentage distribution of the IPLs. IPLs were grouped according to their characteristics as summarized in S3–S5 Tables of the Supporting Information. Colours indicate the sampling months while shade and symbol represent the organ sampled from krill. LMW, low molecular weight; MMW, medium molecular weight; HMW, high molecular weight; LDU, low degree of unsaturation; MDU, medium degree of unsaturation; HDU, high degree of unsaturation.

With respect to the compositions of IPLs, differences between krill sampled in summer and autumn were examined by applying principal component analysis using the relative abundances of the 127 identified molecular formulas (Fig 3). For a better visualization and overview of general trends, IPLs were grouped within their subclasses according to their molecular weight and degree of unsaturation as defined in S3–S5 Tables. As this classification was conducted individually for each head group, the boundaries between the groups differ for each IPL class. However, it was confirmed with a PCA conducted for all IPLs individually that the trends observable in the PCA were not altered by pooling multiple lipids into one group.

In the PCA plot, the distribution of digestive gland samples taken during Antarctic summer was more driven by medium and high molecular weight IPLs and by those containing fatty acids with a higher degree of unsaturation compared to stomach and hind gut samples driven by low molecular weight IPLs. These trends are comparable and homogenous for krill acquired in late summer (January and March). Autumn samples (May) showed a more complex composition and are therefore scattered across the PCA plot. In contrast to summer samples, lower molecular weight IPLs with a low degree of unsaturation are responsible for the shifts present in stomachs and hind guts taken in autumn. However, even though they are distributed across the PCA plot, digestive gland samples are clearly separated from the other organs and affected by higher molecular weight and more unsaturated IPLs in autumn as well.

The fatty acid composition of krill is influenced by food quantity and composition, in terms of e.g. phytoplankton classes [29, 48, 49], so that variations in fatty acid composition between seasons are to be expected. Field studies documented that especially the proportion of DHA and EPA in the fatty acid fingerprint of krill increases during spring and summer [6, 11]. Even though our data set is not as extensive as in those studies, these findings are in accordance with our results, as these PUFAs are predominantly responsible for the trends seen in digestive gland samples. However, the accompanying increase of the molecular weight of IPLs due to elongated fatty acid side chains has not been documented so far. Additionally, it is notable that these trends are mostly imprinted in the digestive gland and not as dominant in the other parts of the digestive system or in the organ-free tissue samples as shown in S1 Fig of the Supporting Information. Tissue samples follow the same trend as samples of the hind gut and stomach, underlining the importance of storage of high molecular weight IPLs in the digestive gland, which has already been shown to be an important site for accumulation and modification of PUFAs [28].

**Fig 4:**
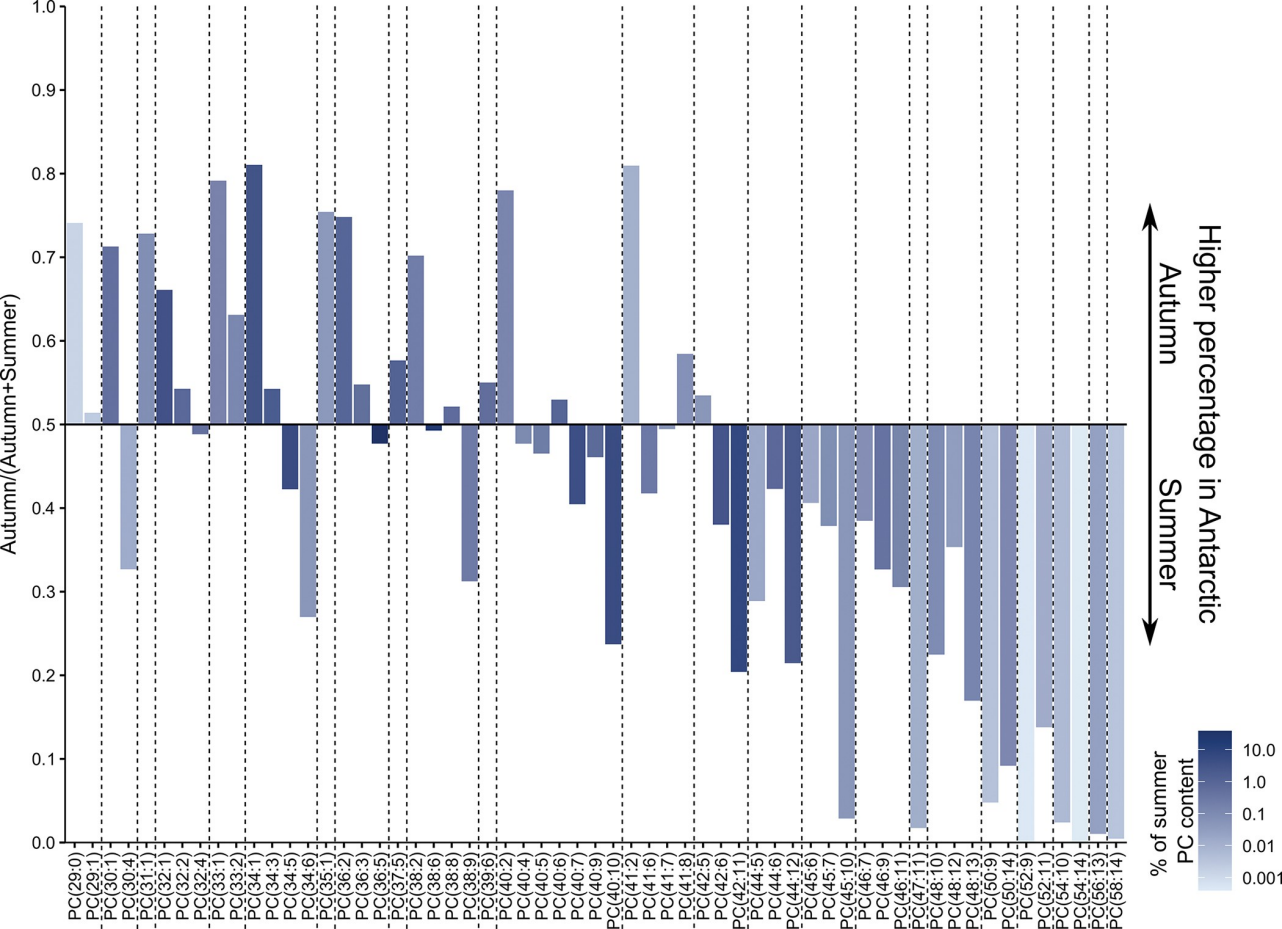
Comparison of the PC composition between Antarctic summer and autumn. Values given reflect the ratio of the percentage distribution of PCs. Lipids represented by bars pointing upwards are more abundant during Antarctic autumn, whereas those pointing downwards are more abundant during Antarctic summer. Colour intensity reflects the percentage of the PCs in summer samples on a logarithmic scale.

As phospholipids containing VLCPUFAs are a main driver for compositional differences observed between months, we directly compared the relation of the IPLs in autumn (May) and summer (January and March) samples. Because PCs are the IPL class with the highest diversity and number of fatty acid combinations, their variation is depicted in Fig 4.

Two distinct features can be observed when comparing the data sets in this way. Firstly, higher molecular weight PCs are more abundant during Antarctic summer. Secondly, within the same chain length of a given PC, the abundance shifts from an increased concentration of more unsaturated PCs during Antarctic summer towards a higher representation of lipids with a low degree of unsaturation in Antarctic autumn. This reflects the increased presence of VLCPUFAs during January and March also seen in the PCA plot.

**Fig 5:**
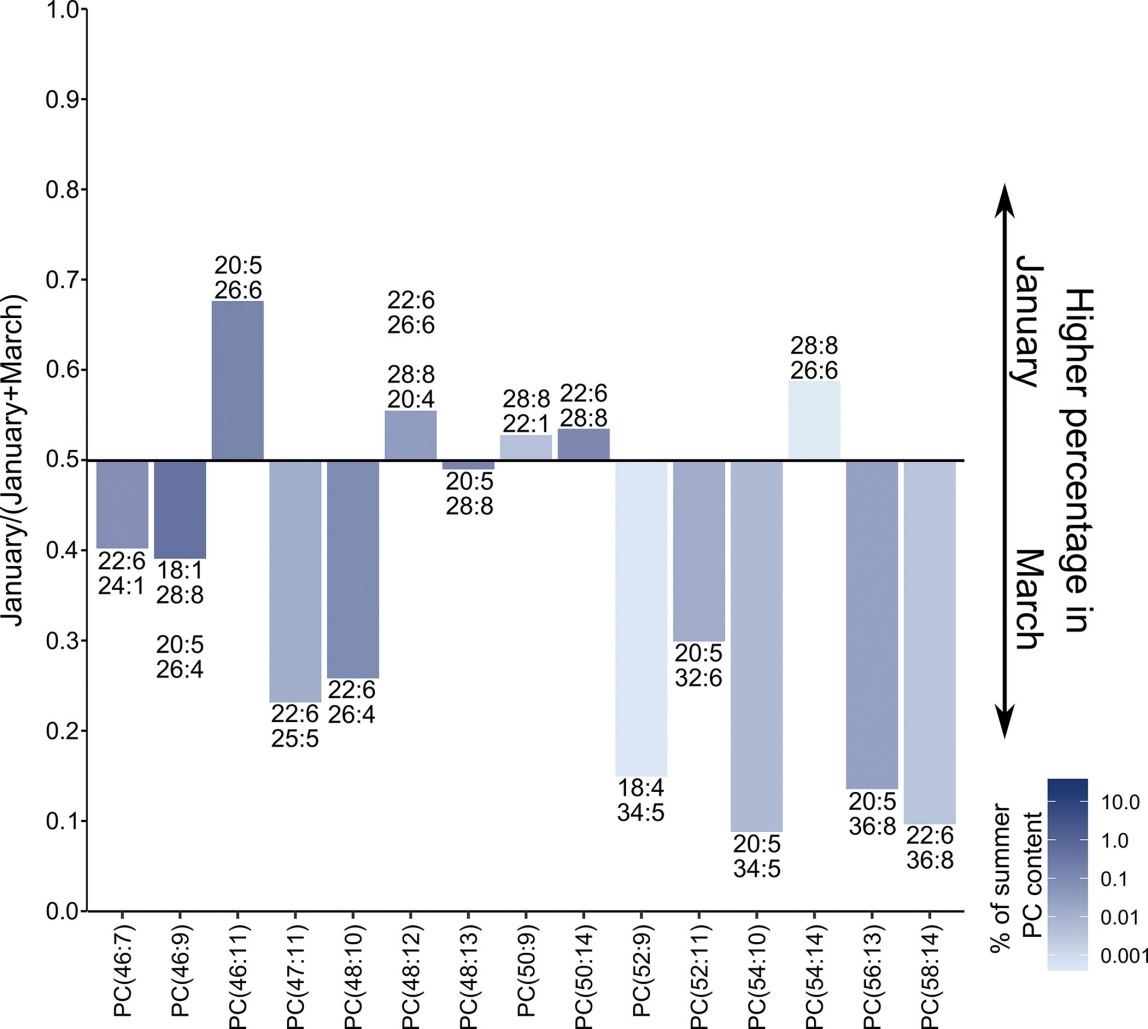
Compositional comparison of the 15 PCs with the highest molecular weight between January and March. Values given reflect the ratio of the percentage distribution of PCs between months. Lipids represented by bars pointing upwards are more abundant in January, whereas those pointing downwards are more abundant in March. Colour intensity reflects the percentage of the PCs in summer samples on a logarithmic scale in accordance with Fig 4. Labels on bars depict the fatty acid composition of the PCs, with the fatty acid of higher intensity in ESI- experiments provided first. For PC(46:9) and PC(48:12) two different combinations were possible, with the more abundant pairing listed first.

Comparing the samples from early and late summer, we observed only minor differences in PCs ranging up to a total of 45 carbon atoms for the two fatty acids combined (S2 Fig). However, there was an increased abundance of high molecular weight PCs in March, except for PCs containing fatty acids 26:6 and 28:8, which were more abundant in January (Fig 5).

Besides an undefined polyunsaturated C_26_ fatty acid, 26:6 has not been described to be present in krill [9]. This fatty acid has been found in tuna oil, in Baltic herrings and in deep-sea brittle stars, even though a pathway for the biosynthesis and not an accumulation due to food intake was proposed for the latter [50–52]. Therefore, the origin of this PUFA remains unknown for now. In contrast, 28:8 has been found in dinoflagellates and may be a biomarker for the utilization of this species by krill [43, 44]. This may lead to the assumption that the dinoflagellate consumption in January was higher than in March.

### Variations in fatty acid compositions between summer and autumn

**Fig 6:**
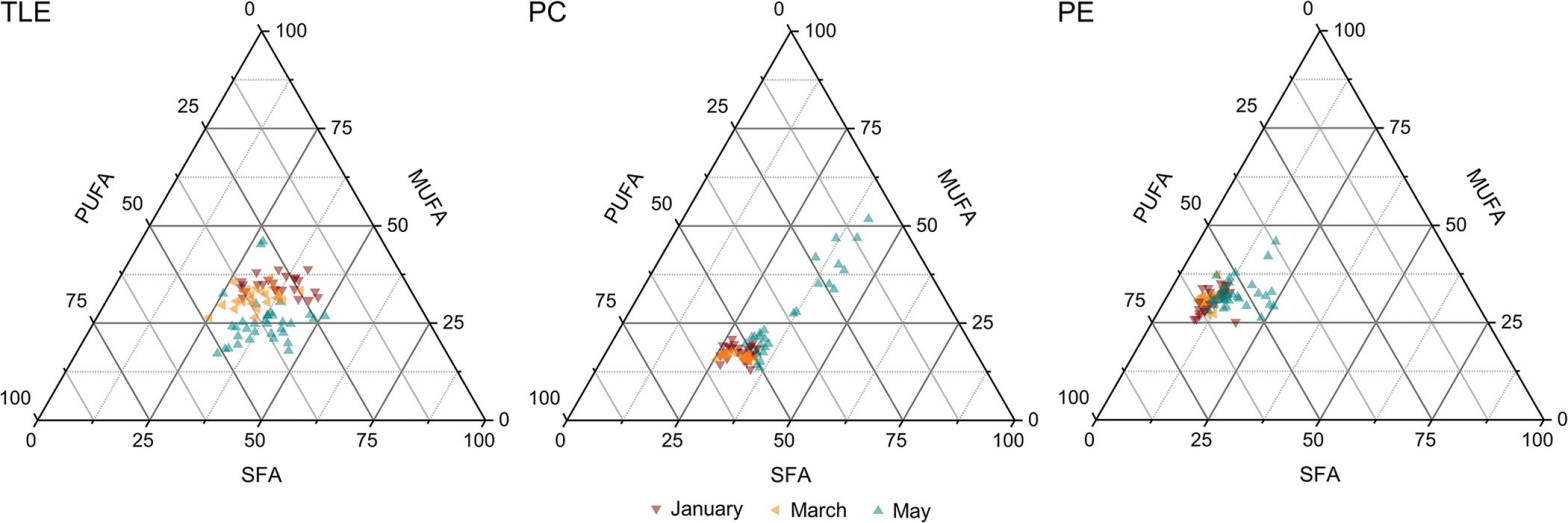
Ternary diagrams of fatty acid compositions. Saturated (SFA), monounsaturated (MUFA) and polyunsaturated (PUFA) fatty acid compositions in the total lipid extracts (TLE) were measured with GC-MS and in phosphatidylcholines (PCs) and phosphatidylethanolamines (PEs) measured with LC-MS. In cases were multiple fatty acid combinations for a given intact phospholipid were observed, the ratio was estimated based on the intensity distribution of the more abundant fatty acid signal in ESI- MS^2^ experiments. All fatty acids listed in S2–S4 Tables were taken into account for the corresponding ternary diagrams.

Rather than comparing individual fatty acid biomarkers described in literature, we evaluated the overall fatty acid composition based on the proportion of saturated, monounsaturated and polyunsaturated fatty acids between the three sampling months. As we did not perform an additional fractionation of the total lipid extract into the different lipid classes, we used the composition of the total lipid extract measured with GC-MS and compared it to the extrapolated fatty acid compositions of the two dominant IPL classes, PCs and PEs, measured with LC-MS (Fig 6).

VLCPUFAs are discriminated during GC-MS measurements, which can have an influence on the results shown in Fig 6. However, the abundance of EPA alone, which was calibrated for GC-MS, accounted for 30 to 40% of the fatty acids in the PC fraction, whereas it was mostly between 10 and 25% in the TLE. Therefore, even when considering a measurement bias, we can observe an increased PUFA content in PCs and PEs, corresponding with published data stating the predominant storage of PUFAs in the IPLs [12, 41, 53].

The summer samples showed a higher MUFA content in the TLEs than the autumn samples, mainly caused by the enhanced 16:1(*n*-7) and 18:1(*n*-7) percentages, which both point towards phytoplankton consumption [49, 54]. This trend was not reflected in the PC and PE compositions, indicating that the cause for this shift must result from the composition of one of the other lipid classes. To imprint such a prominent effect on the composition, the responsible lipid class has to be as dominant as the PCs. Therefore, this shift is potentially caused by the fatty acid composition of triacylglycerols, as those are also very abundant in krill and higher in their MUFA content [10, 12]. A higher proportion of PUFAs in the TLE of summer samples, as reported before [11], could not be observed in this data set. However, the authors documented a high variability in the fatty acid composition of samples taken in autumn, which is also seen in our data. For IPLs, autumn samples had a lower PUFA/SFA ratio. In PCs, eleven autumn samples displayed a PUFA content below 40%. Most of these were stomach samples. The same samples showed a similar trend in PEs, even though the shift was not as distinct as for the PCs.

Fatty acid distributions in organ-free tissue samples also displayed a higher variation in autumn samples for PEs and PCs, potentially hinting at a more versatile diet during this season (S3 Fig). Additionally, the proportion of PUFAs was generally lower than in the digestive system, conversely displaying a higher percentage of SFAs and, at least for the TLE fractions, of MUFAs. Reported abundances of PUFAs in the TLE from digestive gland samples taken in Antarctic summer fall anywhere between 25% and 50% [13, 28, 29]. Even though our data is within this range with 35% for January and 41% for March, this high variability does not facilitate any interpretation. A potential reason for this is the high temporal difference between the four studies (1991 to 2022), since interannual fluctuations of the fatty acid composition already occurs in succeeding years [11].

As differences in the fatty acid composition of phospholipid subclasses have not yet been investigated in other studies, the variation between PCs and PEs as they are depicted in Fig 6 might hint at the different sources of these IPLs. PEs are the dominant IPL in bacteria, which might be a reason for the compositional differences [55, 56]. The increased MUFA content in this subclass supports this assumption.

### Comparison with the Southern Ocean lipidome and future implications

Recently, Holm et al. evaluated the planktonic lipidome across the global ocean in relation to sea surface temperatures with special focus set on the degree of unsaturation and EPA content [57]. One subset of samples originated from a cruise conducted at the West Antarctic Peninsula, opening the possibility for the comparison of PC and PE data from this region to the krill data in our study. As krill mostly feed in the upper ocean but also have been observed migrating to abyssal depth to forage at the sea bed, we took all samples from this study site documented by Holm et al. into consideration, reaching a depth of 254 m [57, 58]. Table 2 shows the diversity in PC and PE sum formulas as well as the corresponding abundances of the overlapping lipids.

**Table 2:** Comparison of PC and PE diversity and abundance between this study and data provided in Holm et al. [57].

	PCs	PEs
Number of sum formulas in this study	59	27
Number of sum formulas in Holm et al.	26	36
Overlap in sum formulas	14	13
Abundance of overlapping sum formulas in this study	45.7%	91.2%
Abundance of overlapping sum formulas in Holm et al.	63.4%	49.3%

Not unexpectedly, sum formulas occurring in both data sets made up a high proportion of the overall abundance in these IPL subclasses. However, over 50% of PC abundances in krill are not represented by the PCs detected by Holm et al. and thus seem to have an origin other than the phytoplankton reflected in those samples, whereas the PE fraction is almost completely covered by the data given in Holm et al. [57]. As PEs are mainly derived from heterotrophic bacteria, this could either hint at bacteria ingested together with other food sources or may reflect the lipid composition of bacteria residing in the digestive system of the krill compared to the surrounding habitat [55, 56].

**Fig 7:**
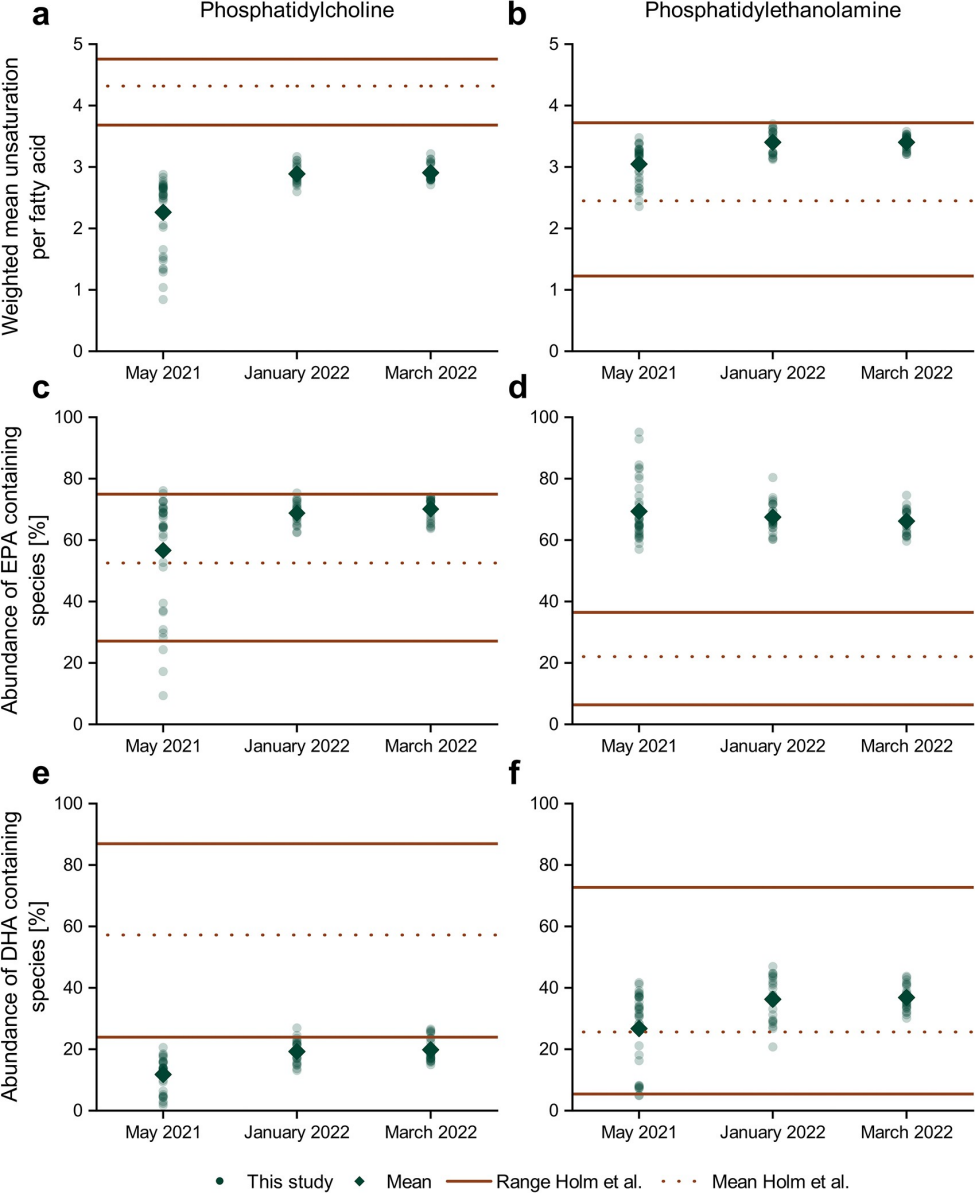
Degree of unsaturation and abundance of EPA and DHA in PCs and PEs. Comparison of the weighted mean of unsaturation per fatty acid (a and b) as well as the percentage of EPA- (c and d) and DHA-containing IPL species (e and f) for PCs (a, c and e) and PEs (b, d and f) between the planktonic lipidome at the Antarctic Peninsula derived from Holm et al. [57] (orange lines, upper and lower limit; dashed line, mean value) and krill data presented in this study (green dots; mean values displayed as diamonds).

Two distinct features evaluated in the study conducted by Holm et al. [57] were the degree of unsaturation within different IPL subclasses as well as the abundance of EPA and DHA. This data was also compared to the krill-derived information (Fig 7).

Contrary to what was expected, krill seems to be depleted in polyunsaturated fatty acids in their PC signature, while unsaturation of PEs fell within the ranges given in Holm et al. [57]. This could hint at a decreased absorption of highly unsaturated IPLs or fatty acids or reflect a discrepancy to planktonic particles, which are not ingested by krill but have contributed to the values in the study of Holm et al. [57].

Despite a high variation in the percentage abundance of EPA-containing PC species in the Antarctic samples evaluated by Holm et al., krill samples plot in the upper section, indicating enrichment of this fatty acid. Considering the lower total unsaturation in PCs, this is a very interesting fact, documenting the importance of EPA in krill, with an even more prominent trend in the PE fraction. For example, juvenile female krill need a high EPA content for their reproductivity, which is hindered in late winter when EPA levels drop [59]. In contrast to this, the abundance of DHA in PCs was rather low, falling below the ranges shown in Holm et al. The opposing trends seen for EPA and DHA are in accordance with data acquired from macrozooplankton, where differences in the requirements of these fatty acids was suggested [60].

Samples of the planktonic lipidome were taken during Antarctic spring of 2018, a year in which phytoplankton biomass was high and diatoms were dominating the phytoplankton composition at the West Antarctic Peninsula [61]. As EPA is considered a biomarker for diatom ingestion, this makes the comparably higher abundances of EPA in krill in our dataset even more intriguing. It further hints at the conversion of other fatty acids into PUFAs and especially EPA, which was already postulated by Virtue et al. [28].

Holm et al. predicted a substantial relative loss of planktonic EPA until the end of the century in particular in the higher latitudes, although more pronounced in the northern than in the southern hemisphere. It has already been documented that a lower proportion of diatoms and flagellates in the krill’s diet will result in a decrease in its EPA content [62]. Estimated shifts in phytoplankton abundances and communities will further alter the biochemical composition of krill and will subsequently have an effect on organisms of higher trophic level [4, 63, 64]. Therefore, future alterations in the Southern Ocean caused by climate change will have a lasting impact on the food chain, starting at the lowest trophic level [5]. These effects will also influence the nutritional benefit of krill and therefore affect krill fishery.

The medical benefit of krill-derived EPA and DHA as nutritional supplement has been widely recognized and is steadily gaining in interest [65, 66]. If krill fishing rates have to be reduced due to decreasing stocks or if EPA and DHA levels drop, other krill-derived omega-3 fatty acids may be additionally tested in order to increase potential use of fatty acids extracted from krill. Decreased levels of VLCPUFAs in humans where demonstrated to be a source of eye-related diseases such as Stargardt-like macular dystrophy type 3 and age-related macular degeneration, while the supplementation with VLCPUFAs resulted in the improvement of visual functions in mice [67–69]. Therefore, nutritional supplement of krill derived VLCPUFAs may add another benefit to the list of krill-derived products. As we identified those especially in krill samples taken in March, krill fishery in summer may yield higher quality krill oil inheriting a greater nutritional benefit.

## Conclusion

As a major constituent and storage lipid of krill, phospholipids are impacted by the nutrient availability in the Southern Ocean, directly reflecting changes occurring in the lowest trophic level. However, fatty acid fingerprints acquired by the analysis of hydrolysed lipids do not fully depict the compositional complexity as it is present in the IPLs. The advantage of high-resolution mass spectrometry to detect VLCPUFAs previously not identified in krill without any discrimination during measurements as demonstrated in this study will provide a valuable tool to monitor variances caused by dynamic changes in the biogeochemical cycle. Even though an expanded temporal resolution and information on spatial variations will be interesting for future investigations, we offer the first data set documenting changes in IPL compositions between summer and autumn. Our data shows that summer samples and in particular samples from the digestive gland where especially enriched in IPLs containing high molecular weight fatty acids with a high degree of unsaturation, which could be linked to the consumption of dinoflagellates. Including these previously overlooked fatty acids into the biomarker portfolio can complement the interpretation possibilities for future studies on the biochemical composition of krill.

## Supporting information

S1 Table Orbitrap settings. Settings used for high-resolution mass spectrometry on the Orbitrap instrument.
(PDF)

S2 Table Free fatty acids of the total lipid extract identified during GC-MS measurements. Fatty acids marked in bold were identified with authentic standards.
(PDF)

S3 Table Phosphatidylcholins. Detected intact phosphatidylcholins (PC), their fatty acid compositions and exact high-resolution masses measured with positive and negative electrospray ionization. The more abundant fatty signal is listed first.
(PDF)

S4 Table Phosphatidylethanolamines. Detected intact phosphatidylethanolamines (PE), phosphatidyl-*N*-methylethanolamines (PME), Phosphatidyl-*N*,*N´*-dimethylethanolamines (PDME) and phosphatidylserines (PS), their fatty acid compositions and exact high-resolution masses measured with positive and negative electrospray ionization. The more abundant fatty signal is listed first.
(PDF)

S5 Table Phosphatidylglycerols and phosphatidylinositides. Detected intact phosphatidylglycerols (PG) and phosphatidylinositides (PI), their fatty acid compositions and exact high-resolution masses measured with positive and negative electrospray ionization. The more abundant fatty signal is listed first.
(PDF)

S1 Fig PCA including tissue samples. Principal component analysis of the percentage distribution of the IPLs grouped according to their characteristics as summarized in S2–S4 Tables. Colours indicate the sampling months while shades and symbols represent the organ sampled from krill as indicated in the legend on the top left corner, including organ-free tissue samples. LMW low molecular weight, MMW medium molecular weight, HMW high molecular weight, LDU low degree of unsaturation, MDU medium degree of unsaturation, HDU high degree of unsaturation.
(PDF)

S2 Fig Compositional comparison of PC(29:0) to PC(45:7) between January and March. Values given reflect the ratio of the percentage distribution of PCs between months. Lipids represented by bars pointing upwards are more abundant in January, whereas those pointing downwards are more abundant in March. Colour intensity reflects the percentage of the PCs in summer samples in a logarithmic scale in accordance with Fig 4 in the main text.
(PDF)

S3 Fig Ternary diagrams of tissue samples. Ternary diagrams of the saturated (SFA), monounsaturated (MUFA) and polyunsaturated (PUFA) fatty acid composition in the total lipid extracts (TLEs), PCs and PEs in organ-free tissue samples. In cases were multiple fatty acid combinations for a given IPL were observed, the ratio was estimated based on the intensity distribution of the more abundant fatty acid signal in ESI- MS^2^ experiments.
(PDF)
