# Supplementary material for: Variations of intact phospholipid compositions in the digestive system of Antarctic krill, Euphausia superba, between summer and autumn
Source: PLoS One. 2023 Dec 29;18(12):e0295677. doi: 10.1371/journal.pone.0295677 (PMC10756546; doi:10.1371/journal.pone.0295677)
Supplement: S1 Table — Settings used for high-resolution mass spectrometry on the Orbitrap instrument. (PDF) [file pone.0295677.s001.pdf]

S1 Table. Settings used for high-resolution mass spectrometry on the Orbitrap instrument.

	ESI+ Full Scan		ESI+ MS^2			ESI- MS^2	
Retention Time (min)	0 – 43.7	3.5 - 5	5 - 8	8 - 9	3.5 - 5	5 - 8	8 - 9
IPL		PG	PE, PME, PDME, PI	PC, PS	PG	PE, PME, PDME, PI	PC, PS
Spray Voltage (V)	3000	4000	3600	2100	-3400	-3800	-3600
Sheath Gas (Arb)	30		25			24	
Aux Gas (Arb)	10		10			13	
Sweep Gas (Arb)	1		1			1	
Ion Transfer Tube Temperature (°C)	285		285			285	
Vaporizer Temperature (°C)	320		320			320	
Orbitrap Resolution	240 000		60 000			60 000	
Scan Range	350 - 1400		350 - 1600			350 - 1600	
RF Lens (%)	45		45			45	
AGC Target	2.00E+05		4.00E+05			4.00E+05	
Maximum Injection Time (ms)	50		50			50	
			MS^2 settings			MS^2 settings	
Intensity Threshold			2.50E+04			2.50E+04	
Isolation Window (*m/z*)			1			1	
Isolation Offset			Off			Off	
Activation Type			HCD			HCD	
Collision Energy Mode			Stepped			Stepped	
HCD Collision Energies (%)		0, 15, 20	10, 20, 25	5, 25, 30	10, 30, 40	10, 25, 35	0, 20, 30
Orbitrap Resolution			30 000			30 000	
First Mass (*m/z*)			100			100	
AGC Target			6.04E+04			6.04E+04	
Maximum Injection Time (ms)			54			54	
