# Supplementary material for: Variations of intact phospholipid compositions in the digestive system of Antarctic krill, Euphausia superba, between summer and autumn
Source: PLoS One. 2023 Dec 29;18(12):e0295677. doi: 10.1371/journal.pone.0295677 (PMC10756546; doi:10.1371/journal.pone.0295677)
Supplement: S2 Table — Fatty acids marked in bold were identified with authentic standards. (PDF) [file pone.0295677.s002.pdf]

S2 Table. Free fatty acids of the total lipid extract identified during GC-MS measurements. Fatty acids marked in bold were identified with authentic standards.

Saturated fatty acids	Calculated retention index	Monounsaturated fatty acids	Calculated retention index	Polyunsaturated fatty acids	Calculated retention index
8:0	1126	xMe16:1	1982	16:4	1887
9:0	1228	14:1	1712	16:3	1894
**10:0**	**1326**	**16:1 (n-7)**	**1905**	16:2	1908
11:0	1421	16:1 (n-5)	1914	PUFA18	2072
**12:0**	**1529**	17:1	2002	18:4	2088
13:0	1630	17:1	2022	18:2	2097
**14:0**	**1725**	**18:1 (n-9)**	**2103**	18:2	2144
15:0	1824	18:1 (n-7)	2109	**20:5**	**2276**
**16:0**	**1927**	18:1 (n-5)	2118	PUFA20	2281
**17:0**	**2026**	20:1	2304	20:4	2290
**18:0**	**2127**	20:1	2310	PUFA20	2297
19:0	2227	**22:1 (n-9)**	**2506**	PUFA21	2375
**20:0**	**2328**	22:1	2513	22:6	2462
**22:0**	**2530**	23:1	2607	PUFA22	2473
12Me13:0	1687	24:1	2710		
13Me14:0	1788				
12Me14:0	1796				
14Me15:0	1883				
13Me15:0	1887				
15Me16:0	1988				
14Me16:0	1998				
16Me17:0	2085				

Nomenclature: 12Me14:0, tetradecanoic acid with an additional methyl group at C12 (anteiso-pentadecanoic acid); xMe, fatty acid with methyl group at unknown position; PUFA, uncharacterised polyunsaturated fatty acid; (n-x), position of double bound.
