# Supplementary material for: Variations of intact phospholipid compositions in the digestive system of Antarctic krill, Euphausia superba, between summer and autumn
Source: PLoS One. 2023 Dec 29;18(12):e0295677. doi: 10.1371/journal.pone.0295677 (PMC10756546; doi:10.1371/journal.pone.0295677)
Supplement: S3 Table — Detected intact phosphatidylcholins (PC), their fatty acid compositions and exact high-resolution masses measured with positive and negative electrospray ionization. The more abundant fatty signal is listed first. (PDF) [file pone.0295677.s003.pdf]

S3 Table. Detected intact phosphatidylcholines (PCs), their fatty acid compositions and exact high-resolution masses measured with positive and negative electrospray ionization. The more abundant fatty signal is listed first.

Phospholipid	Fatty acids	ESI+ $[M+H]^+$ (*m/z*)	ESI- $[M+COO]^-$ (*m/z*)	Phospholipid	Fatty acids	ESI+ $[M+H]^+$ (*m/z*)	ESI- $[M+COO]^-$ (*m/z*)
PC(29:0)[a]	16:0 / 13:0	692.5225	736.5134	PC(39:6)[d]	20:4 / 19:2	820.5851	864.5760
PC(29:1)[a]	16:1 / 13:0	690.5068	734.4978	PC(40:2)[c]	22:1 / 18:1	842.6633	886.6543
PC(29:1)[a]	15:1 / 14:0	690.5068	734.4978	PC(40:4)[c]	18:3 / 22:1	838.6320	882.6230
PC(30:1)[a]	16:1 / 14:0	704.5225	748.5134	PC(40:5)[d]	18:4 / 22:1	836.6164	880.6073
PC(30:1)[a]	14:1 / 16:0	704.5225	748.5134	PC(40:6)[d]	20:5 / 20:1	834.6007	878.5917
PC(30:4)[b]	16:4 / 14:0	698.4755	742.4665	PC(40:7)[d]	22:6 / 18:1	832.5851	876.5760
PC(31:1)[a]	15:0 / 16:1	718.5381	762.5291	PC(40:9)[e]	20:5 / 20:4	828.5538	872.5436
PC(31:1)[a]	14:0 / 17:1	718.5381	762.5291	PC(40:9)[e]	22:6 / 18:3	828.5538	872.5436
PC(32:1)[a]	14:0 / 18:1	732.5538	776.5447	PC(40:10)[e]	20:5 / 20:5	826.5381	870.5291
PC(32:1)[a]	16:0 / 16:1	732.5538	776.5447	PC(41:2)[c]	18:1 / 23:1	856.6790	900.6699
PC(32:2)[a]	16:1 / 16:1	730.5381	774.5291	PC(41:6)[d]	20:5 / 21:1	848.6164	892.6073
PC(32:4)[b]	18:4 / 14:0	726.5068	770.4978	PC(41:7)[d]	22:6 / 19:1	846.6007	890.5917
PC(32:4)[b]	16:4 / 16:0	726.5068	770.4978	PC(41:8)[d]	22:6 / 19:2	844.5851	888.5760
PC(33:1)[a]	18:1 / 15:0	746.5694	790.5604	PC(42:5)[d]	20:5 / 22:0	864.6477	908.6386
PC(33:1)[a]	16:0 / 17:1	746.5694	790.5604	PC(42:5)[d]	18:4 / 24:1	864.6477	908.6386
PC(33:2)[a]	18:1 / 15:1	744.5538	788.5447	PC(42:6)[d]	20:5 / 22:1	862.6320	906.6230
PC(33:2)[a]	16:0 / 17:2	744.5538	788.5447	PC(42:11)[e]	20:5 / 22:6	852.5538	896.5447
PC(33:2)[a]	18:2 / 15:0	744.5538	788.5447	PC(44:5)[d]	20:5 / 24:0	892.6790	936.6699
PC(33:2)[a]	16:1 / 17:1	744.5538	788.5447	PC(44:5)[d]	20:4 / 24:1	892.6790	936.6699
PC(33:2)[a]	14:0 / 19:2	744.5538	788.5447	PC(44:6)[d]	20:5 / 24:1	890.6633	934.6543
PC(34:1)[a]	16:0 / 18:1	760.5851	804.5760	PC(44:12)[e]	22:6 / 22:6	878.5694	922.5604
PC(34:3)[b]	18:3 / 16:0	756.5538	800.5447	PC(45:6)[d]	20:5 / 25:1	904.6790	948.6699
PC(34:5)[b]	16:0 / 22:5	752.5225	796.5134	PC(45:6)[d]	22:5 / 23:1	904.6790	948.6699
PC(34:6)[b]	20:5 / 14:1	750.5068	794.4978	PC(45:7)[d]	22:6 / 23:1	902.6633	946.6543
PC(35:1)[a]	18:1 / 17:0	774.6007	818.5917	PC(45:10)[f]	20:5 / 25:5	896.6164	940.6073
PC(35:1)[a]	16:0 /19:1	774.6007	818.5917	PC(46:7)[f]	22:6 / 24:1	916.6790	960.6699
PC(36:2)[a]	18:1 / 18:1	786.6007	830.5917	PC(46:9)[f]	18:1 / 28:8	912.6477	956.6386
PC(36:3)[b]	18:2 / 18:1	784.5851	828.5760	PC(46:9)[f]	20:5 / 26:4	912.6477	956.6386
PC(36:3)[b]	16:0 / 20:3	784.5851	828.5760	PC(46:11)[f]	20:5 / 26:6	908.6164	952.6073
PC(36:5)[b]	20:5 / 16:0	780.5538	824.5447	PC(47:11)[f]	22:6 / 25:5	922.6320	966.6219
PC(37:5)[b]	20:5 / 17:0	794.5694	838.5604	PC(48:10)[f]	22:6 / 26:4	938.6633	982.6543
PC(37:5)[b]	21:5 / 16:0	794.5694	838.5604	PC(48:12)[f]	22:6 / 26:6	934.6320	978.6230
PC(38:2)[c]	18:1 / 20:1	814.6320	858.6230	PC(48:12)[f]	28:8 / 20:4	934.6320	978.6230
PC(38:2)[c]	16:1 / 22:1	814.6320	858.6230	PC(48:13)[f]	20:5 / 28:8	932.6164	976.6073
PC(38:6)[d]	18:1 / 20:5	806.5694	850.5604	PC(50:9)[f]	28:8 / 22:1	968.7103	1012.7012
PC(38:6)[d]	16:0 / 22:6	806.5694	850.5604	PC(50:14)[f]	22:6 / 28:8	958.6320	1002.6230
PC(38:8)[e]	20:5 / 18:3	802.5381	846.5291	PC(52:9)[f]	18:4 / 34:5	996.7416	1040.7325
PC(38:9)[e]	18:4 / 20:5	800.5225	844.5134	PC(52:11)[f]	20:5 / 32:6	992.7103	1036.7012
PC(39:6)[d]	22:6 / 17:0	820.5851	864.5760	PC(54:10)[f]	20:5 / 34:5	1022.7572	1066.7482
PC(39:6)[d]	20:5 / 19:1	820.5851	864.5760	PC(54:14)[f]	28:8 / 26:6	1014.6946	1058.6856
PC(39:6)[d]	21:5 / 18:1	820.5851	864.5760	PC(56:13)[f]	20:5 / 36:8	1044.7416	1088.7325
PC(39:6)[d]	22:5 / 17:1	820.5851	864.5760	PC(58:14)[f]	22:6 / 36:8	1070.7572	1114.7482

[a-f] Groups used for the PCA analysis, [a] low molecular weight and low degree of unsaturation, [b] low molecular weight and medium degree of unsaturation, [c] medium molecular weight and low degree of unsaturation, [d] medium molecular weight and medium degree of unsaturation, [e] medium molecular weight and high degree of unsaturation, [f] high molecular weight and high degree of unsaturation.
