# Supplementary material for: Variations of intact phospholipid compositions in the digestive system of Antarctic krill, Euphausia superba, between summer and autumn
Source: PLoS One. 2023 Dec 29;18(12):e0295677. doi: 10.1371/journal.pone.0295677 (PMC10756546; doi:10.1371/journal.pone.0295677)
Supplement: S4 Table — Detected intact phosphatidylethanolamines (PE), phosphatidyl-N-methylethanolamines (PME), Phosphatidyl-N,N´-dimethylethanolamines (PDME) and phosphatidylserines (PS), their fatty acid compositions and exact high-resolution masses measured with positive and negative electrospray ionization. The more abundant fatty signal is listed first. (PDF) [file pone.0295677.s004.pdf]

S4 Table. Detected intact phosphatidylethanolamines (PEs), phosphatidyl-*N*-methylethanolamines (PMEs), phosphatidyl-*N*,*N*´-dimethylethanolamines (PDMEs) and phosphatidylserines (PSs), their fatty acid compositions and exact high-resolution masses measured with positive and negative electrospray ionization. The more abundant fatty signal is listed first.

Phospholipid	Fatty acids	ESI+ $[M+H]^+$ (*m/z*)	ESI- $[M-H]^-$ (*m/z*)
PE(32:2)[g]	16:1 / 16:1	688.4912	686.4755
PE(32:2)[g]	18:2 / 14:0	688.4912	686.4755
PE(32:2)[g]	16:2 / 16:0	688.4912	686.4755
PE(32:2)[g]	18:1 / 14:1	688.4912	686.4755
PE(34:1)[g]	18:1 / 16:0	718.5381	716.5236
PE(34:4)[h]	20:4 / 14:0	712.4912	710.4766
PE(34:4)[h]	18:4 / 16:0	712.4912	710.4766
PE(34:5)[h]	14:0 / 20:5	710.4755	708.4599
PE(34:5)[h]	18:4 / 16:1	710.4755	708.4599
PE(34:5)[h]	18:1 / 16:4	710.4755	708.4599
PE(34:6)[h]	20:5 / 14:1	708.4598	706.4442
PE(34:6)[h]	22:6 / 12:0	708.4598	706.4442
PE(35:2)[g]	18:1 / 17:1	730.5381	728.5225
PE(36:2)[g]	18:1 / 18:1	744.5538	742.5381
PE(36:3)[h]	18:2 / 18:1	742.5381	740.5236
PE(36:3)[h]	20:3 / 16:0	742.5381	740.5236
PE(36:5)[h]	16:0 / 20:5	738.5068	736.4912
PE(38:2)[g]	20:1 / 18:1	772.5851	770.5694
PE(38:5)[i]	20:5 / 18:0	766.5381	764.5225
PE(38:5)[i]	20:4 / 18:1	766.5381	764.5225
PE(38:6)[i]	18:1 / 20:5	764.5225	762.5068
PE(38:6)[i]	16:0 / 22:6	764.5225	762.5068
PE(38:8)[i]	20:5 / 18:3	760.4912	758.4766
PE(38:8)[i]	22:6 / 16:2	760.4912	758.4766
PE(38:8)[i]	18:4 / 20:4	760.4912	758.4766
PE(40:6)[i]	18:1 / 22:5	792.5538	790.5381
PE(40:6)[i]	20:1 / 20:5	792.5538	790.5381
PE(40:7)[i]	18:1 / 22:6	790.5381	788.5225
PE(40:9)[j]	20:5 / 20:4	786.5068	784.4923
PE(40:9)[j]	22:6 / 18:3	786.5068	784.4923
PE(40:10)[j]	20:5 / 20:5	784.4912	782.4755
PE(41:7)[i]	19:1 / 22:6	804.5538	802.5381
PE(42:7)[i]	20:1 / 22:6	818.5694	816.5538
PE(42:10)[j]	22:6 / 20:4	812.5225	810.5079
PE(42:10)[j]	20:5 / 22:5	812.5225	810.5079
PE(42:11)[j]	20:5 / 22:6	810.5068	808.4912
PE(44:8)[k]	28:8 / 16:0	844.5851	842.5705
PE(44:8)[k]	26:7 / 18:1	844.5851	842.5705
PE(44:12)[k]	22:6 / 22:6	836.5225	834.5068
PE(46:9)[k]	18:1 / 28:8	870.6007	868.5851
PE(46:11)[k]	20:5 / 26:6	866.5694	864.5538
PE(48:13)[k]	20:5 / 28:8	890.5694	888.5538
PE(50:14)[k]	28:8 / 22:6	916.5851	914.5705
PME(36:5)[l]	20:5 / 16:0	752.5225	750.5068
PME(38:6)[l]	20:5 / 18:1	778.5381	776.5225
PME(38:6)[l]	16:0 / 22:6	778.5381	776.5225
PME(40:7)[l]	18:1 / 22:6	804.5538	802.5381
PME(40:9)[m]	20:5 / 20:4	800.5225	798.5068
PME(40:10)[m]	20:5 / 20:5	798.5068	796.4912
PME(42:11)[m]	20:5 / 22:6	824.5225	822.5068
PME(44:12)[m]	22:6 / 22:6	850.5381	848.5225
PDME(32:1)[n]	16:0 / 16:1	718.5392	716.5225
PDME(32:1)[n]	14:0 / 18:1	718.5392	716.5225
PDME(34:1)[n]	16:0 / 18:1	746.5697	744.5538
PDME(36:2)[n]	18:1 / 18:1	772.5851	770.5694
PDME(36:5)[o]	20:5 / 16:0	766.5381	764.5225
PDME(38:5)[o]	20:5 / 18:0	794.5694	792.5549
PDME(38:6)[o]	18:1 / 20:5	792.5538	790.5381
PDME(38:6)[o]	22:6 / 16:0	792.5538	790.5381
PDME(40:6)[o]	20:5 / 20:1	820.5851	818.5705
PDME(40:7)[o]	18:1 / 22:6	818.5694	816.5549
PDME(40:10)[p]	20:5 / 20:5	812.5225	810.5079
PDME(42:6)[o]	20:5 / 22:1	848.6164	846.6007
PDME(42:11)[p]	20:5 / 22:6	838.5381	836.5236
PDME(43:6)[o]	20:5 / 23:1	862.6320	860.6175
PDME(44:12)[p]	22:6 / 22:6	864.5538	862.5381
PS(36:5)[q]	16:0 / 20:5	782.4967	780.4810
PS(38:6)[q]	18:1 / 20:5	808.5123	806.4967
PS(38:6)[q]	16:0 / 22:6	808.5123	806.4967
PS(39:7)[q]	19:2 / 20:5	820.5123	818.4967
PS(40:7)[q]	18:1 / 22:6	834.5280	832.5123

[g-q] Groups used for the PCA analysis, [g,n] low molecular weight and low degree of unsaturation, [h] low molecular weight and medium degree of unsaturation, [i,l,o,q] medium molecular weight and medium degree of unsaturation, [j,m,p] medium molecular weight and high degree of unsaturation, [k] high molecular weight and high degree of unsaturation.
