# Supplementary material for: Variations of intact phospholipid compositions in the digestive system of Antarctic krill, Euphausia superba, between summer and autumn
Source: PLoS One. 2023 Dec 29;18(12):e0295677. doi: 10.1371/journal.pone.0295677 (PMC10756546; doi:10.1371/journal.pone.0295677)
Supplement: S5 Table — Detected intact phosphatidylglycerols (PG) and phosphatidylinositides (PI), their fatty acid compositions and exact high-resolution masses measured with positive and negative electrospray ionization. The more abundant fatty signal is listed first. (PDF) [file pone.0295677.s005.pdf]

S5 Table. Detected intact phosphatidylglycerols (PGs) and phosphatidylinositides (PIs), their fatty acid compositions and exact high-resolution masses measured with positive and negative electrospray ionization. The more abundant fatty signal is listed first.

Phospholipid	Fatty acids	ESI+ $[M+NH_4]^+$ (*m/z*)	ESI- $[M-H]^-$ (*m/z*)
PG(34:1)[r]	16:0 / 18:1	766.5593	747.5171
PG(36:1)[r]	20:1 / 16:0	794.5906	775.5484
PG(36:2)[r]	16:0 / 20:2	792.5749	773.5327
PG(36:5)[s]	16:0 / 20:5	786.5280	767.4858
PG(38:6)[s]	18:1 / 20:5	812.5436	793.5014
PG(40:10)[t]	20:5 / 20:5	832.5123	813.4701
PI(35:5)[u]	20:5 / 15:0	860.5284	841.4862
PI(36:5)[u]	16:0 / 20:5	874.5440	855.5018
PI(37:4)[u]	21:4 / 16:0	890.5753	871.5331
PI(37:4)[u]	20:4 / 17:0	890.5753	871.5331
PI(37:5)[u]	20:5 / 17:0	888.5597	869.5175
PI(37:6)[u]	22:6 / 15:0	886.5440	867.5018
PI(37:6)[u]	20:5 / 17:1	886.5440	867.5018
PI(38:5)[u]	18:1 / 20:4	902.5753	883.5331
PI(38:5)[u]	20:5 / 18:0	902.5753	883.5331
PI(38:5)[u]	16:0 / 22:5	902.5753	883.5331
PI(38:6)[u]	18:1 / 20:5	900.5597	881.5175
PI(38:6)[u]	16:0 / 22:6	900.5597	881.5175
PI(38:7)[u]	18:2 / 20:5	898.5440	879.5018
PI(38:7)[u]	18:3 / 20:4	898.5440	879.5018
PI(38:8)[u]	18:3 / 20:5	896.5284	877.4862
PI(39:5)[u]	18:1 / 21:4	916.5910	897.5488
PI(39:5)[u]	20:5 / 19:0	916.5910	897.5488
PI(40:7)[u]	18:1 / 22:6	926.5753	907.5331

[r-u] Groups used for the PCA analysis, [r] low molecular weight and low degree of unsaturation, [s] low molecular weight and medium degree of unsaturation, [u] medium molecular weight and medium degree of unsaturation, [t] medium molecular weight and high degree of unsaturation.
