# Supplementary material for: Variations of intact phospholipid compositions in the digestive system of Antarctic krill, Euphausia superba, between summer and autumn
Source: PLoS One. 2023 Dec 29;18(12):e0295677. doi: 10.1371/journal.pone.0295677 (PMC10756546; doi:10.1371/journal.pone.0295677)
Supplement: S1 Fig — Principal component analysis of the percentage distribution of the IPLs grouped according to their characteristics as summarized in S2–S4 Tables. Colours indicate the sampling months while shades and symbols represent the organ sampled from krill as indicated in the legend on the top left corner, including organ-free tissue samples. LMW low molecular weight, MMW medium molecular weight, HMW high molecular weight, LDU low degree of unsaturation, MDU medium degree of unsaturation, HDU high degree of unsaturation. (PDF) [file pone.0295677.s006.pdf]

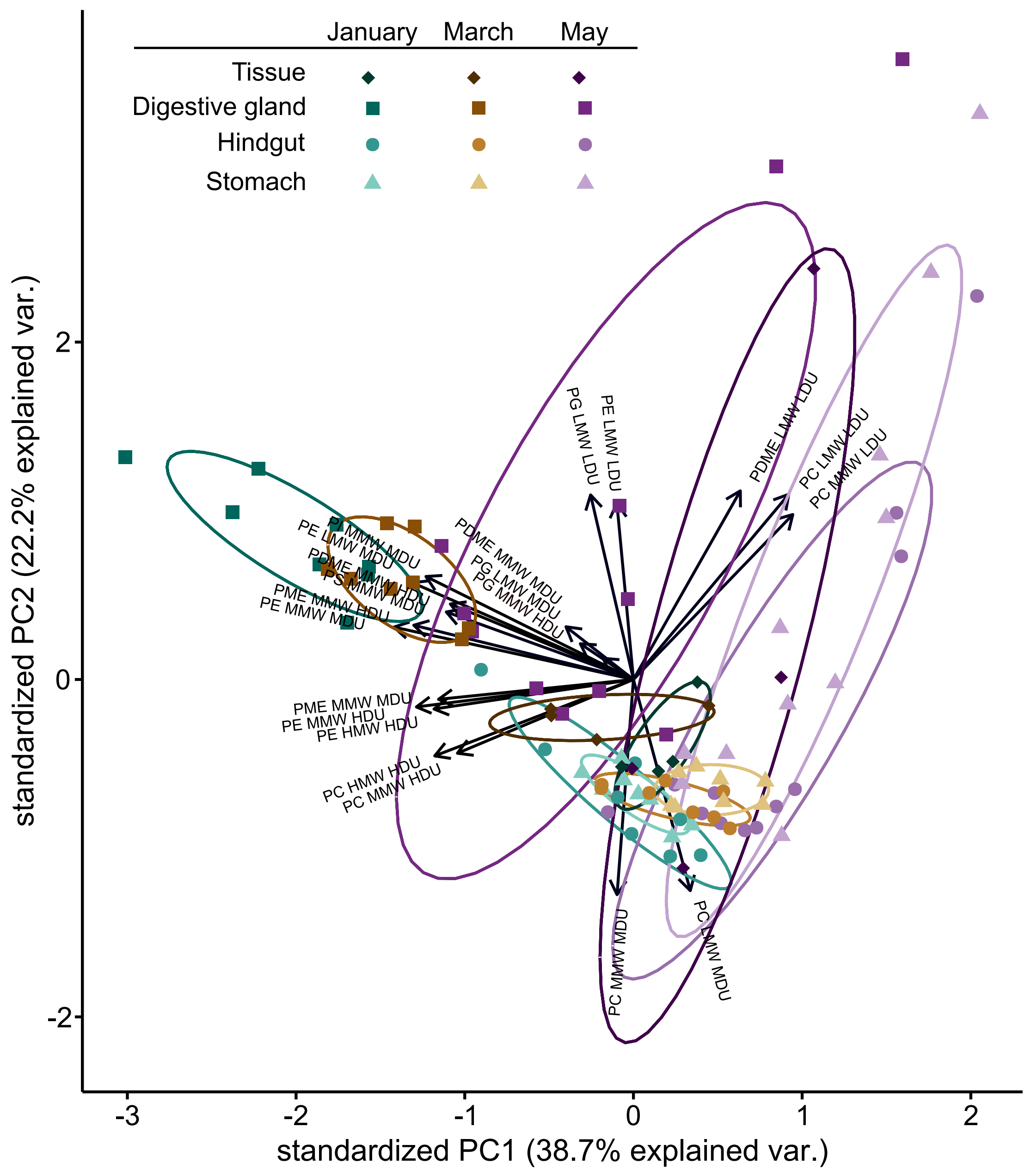

S1 Fig. Principal component analysis of the percentage distribution of the IPLs grouped according to their characteristics as summarized in tables S2-4. Colours indicate the sampling months while shades and symbols represent the organ sampled from krill as indicated in the legend on the top left corner, including organ-free tissue samples. LMW, low molecular weight; MMW, medium molecular weight; HMW, high molecular weight; LDU, low degree of unsaturation; MDU, medium degree of unsaturation; HDU, high degree of unsaturation.
