# Supplementary material for: Variations of intact phospholipid compositions in the digestive system of Antarctic krill, Euphausia superba, between summer and autumn
Source: PLoS One. 2023 Dec 29;18(12):e0295677. doi: 10.1371/journal.pone.0295677 (PMC10756546; doi:10.1371/journal.pone.0295677)
Supplement: S2 Fig — Values given reflect the ratio of the percentage distribution of PCs between months. Lipids represented by bars pointing upwards are more abundant in January, whereas those pointing downwards are more abundant in March. Colour intensity reflects the percentage of the PCs in summer samples in a logarithmic scale in accordance with Fig 4 in the main text. (PDF) [file pone.0295677.s007.pdf]

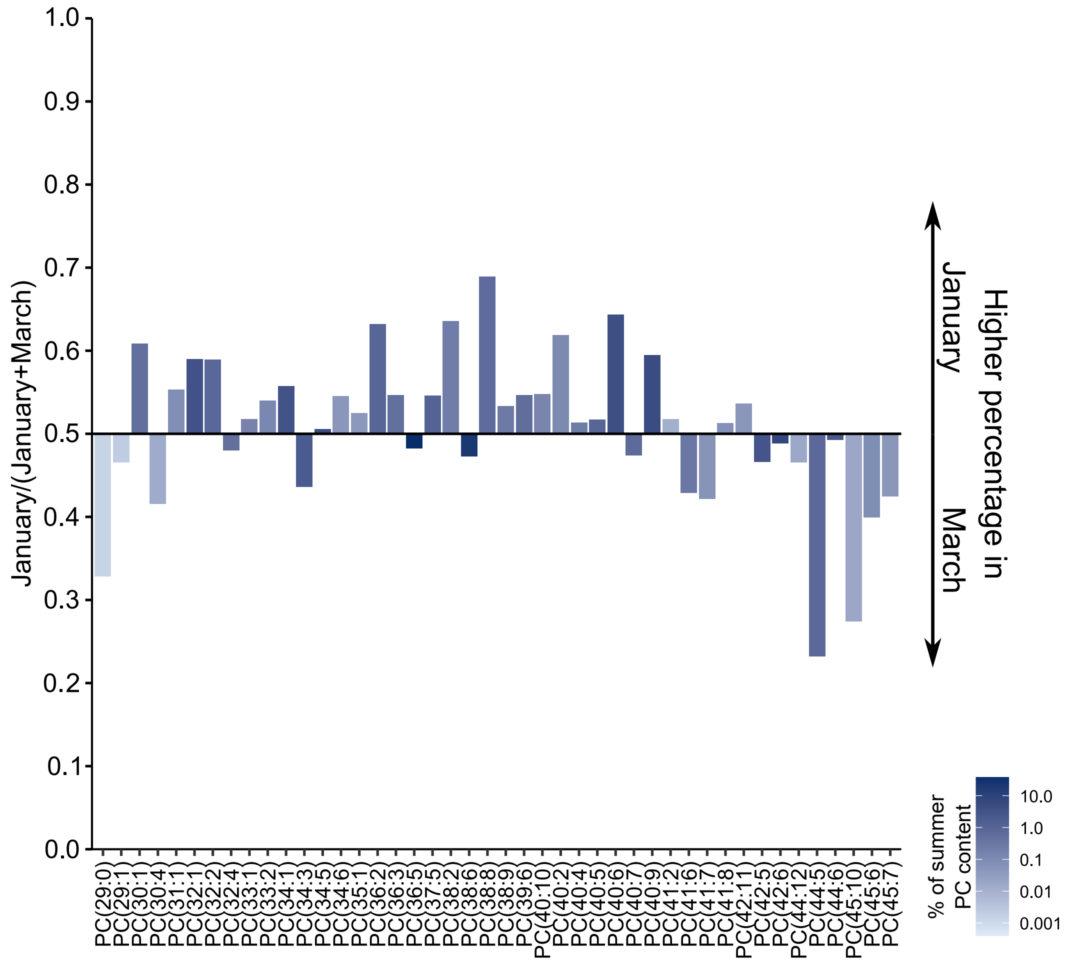

S2 Fig:Compositional comparison of PC(29:0) to PC(45:7)between January and March. Values given reflect the ratio of the percentage distribution of PCs between months. Lipids represented by bars pointing upwards are more abundant in January, whereas those pointing downwards are more abundant in March. Colour intensity reflects the percentage of the PCs in summer samples in a logarithmic scale in accordance with Fig4in the main text.
