# Supplementary material for: Variations of intact phospholipid compositions in the digestive system of Antarctic krill, Euphausia superba, between summer and autumn
Source: PLoS One. 2023 Dec 29;18(12):e0295677. doi: 10.1371/journal.pone.0295677 (PMC10756546; doi:10.1371/journal.pone.0295677)
Supplement: S3 Fig — Ternary diagrams of the saturated (SFA), monounsaturated (MUFA) and polyunsaturated (PUFA) fatty acid composition in the total lipid extracts (TLEs), PCs and PEs in organ-free tissue samples. In cases were multiple fatty acid combinations for a given IPL were observed, the ratio was estimated based on the intensity distribution of the more abundant fatty acid signal in ESI- MS2 experiments. (PDF) [file pone.0295677.s008.pdf]

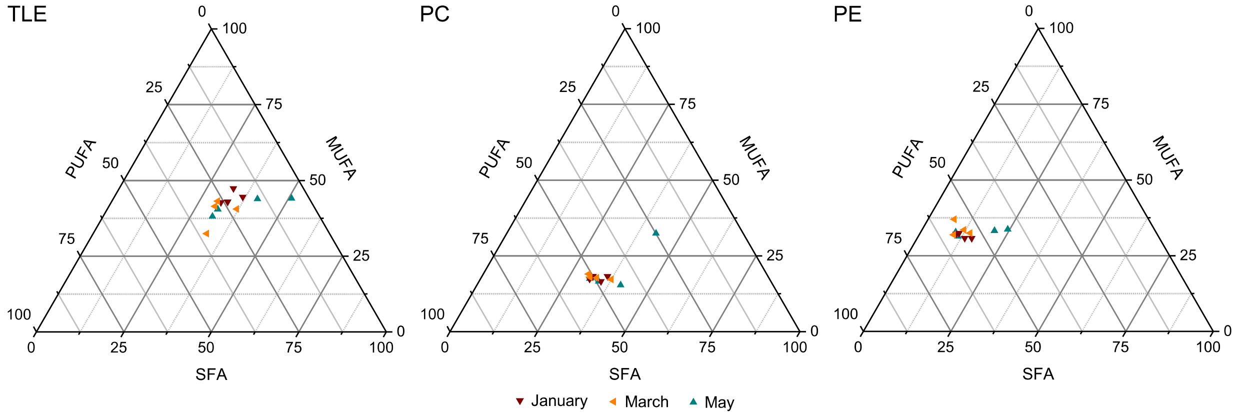

S3 Fig: Ternary diagrams of the saturated (SFA), monounsaturated (MUFA) and polyunsaturated (PUFA) fatty acid composition in the total lipid extracts (TLEs), PCs and PEs in organ-free tissue samples. In cases were multiple fatty acid combinations for a given IPL were observed, the ratio was estimated based on the intensity distribution of the more abundant fatty acid signal in ESI- MS^2 experiments.
